# Inhibiting EMT, stemness and cell cycle involved in baicalin-induced growth inhibition and apoptosis in colorectal cancer cells

**DOI:** 10.7150/jca.37242

**Published:** 2020-02-10

**Authors:** Bolin Yang, Huiru Bai, Yunli Sa, Ping Zhu, Ping Liu

**Affiliations:** 1 Department of Colon and Rectum Surgery, Jiangsu Province Hospital of Chinese Medicine, Affiliated Hospital of Nanjing University of Chinese Medicine, Nanjing, Jiangsu 210029, P R China; 2 Jiangsu Key Laboratory for Molecular and Medicine Biotechnology, College of Life Sciences, Nanjing Normal University, Nanjing, Jiangsu 210023, P R China

**Keywords:** baicalin, EMT, stemness, apoptosis, cell cycle, colorectal cancer (CRC)

## Abstract

Although baicalin, a flavonoid derived from *Scutellaria baicalensis Georgi*, has been reported to have anti-tumor activity in various cancers, the molecular mechanism remains imperfect. Here, we show that baicalin inhibits cell growth, migration and invasion and induces cell apoptosis by inhibiting cell cycle, viability, the epithelial-mesenchymal transition (EMT) and cellular stemness in colorectal cancer (CRC) cells. In detail, baicalin treatment in CRC cells induces cell cycle arrest in G1 phase and promotes p53-independent cell apoptosis, inhibits both endogenous and exogenous TGFβ1-induced EMT of colorectal cancer cells by inhibiting TGFβ/Smad pathway. Cell sphere-formation experiments show that baicalin has a strong inhibitory efficacy on the stemness of CRC cells by decreasing the marker proteins of cancer stem cell (CSC) and inhibits the formation of CSC-like cell spheres in CRC cells. *In vivo* experiments also identify that baicalin has an anti-tumor effect by down-regulating the levels of marker proteins of cell cycle, EMT and stemness in the orthotopic transplantation tumors of CRC cells in BALB/c nude mice. Collectively, our *in vitro* and *in vivo* results indicate that multiple inhibition of cell cycle, EMT and stemness is the real molecular mechanism of baicalin in effectively inducing cell growth inhibition and apoptosis in CRC cells.

## Introduction

Colorectal cancer (CRC) is diagnosed the third most common cancer and the fourth leading cause of cancer-related mortality worldwide 1-3. Although current therapeutic methods such as surgery, chemotherapy, radiotherapy and biotherapy have efficacy, the five-year overall survival remains low due to its strong chemoresistance, high aggressiveness and recurrence 4-7.

Epithelial to mesenchymal transition (EMT) is considered as a pivotal developmental program that is activated frequently during cancer metastasis and invasion 8, 9. Epithelial cells lose their epithelial features (such as down-regulation of E-cadherin), gain mesenchymal phenotypes (such as up-regulation of N-cadherin and Vimentin) and activate some transcription factors (such as Snail, Slug, Twist) in EMT 10-12. Cancer cells undergoing EMT acquire the capacity to migrate and invade into the surrounding stroma and subsequently spread via blood and lymphatic vessels to distant sites 13. Cancer cells have some stem cell-like characteristics (stemness) during EMT, which results in colony formation and expression of stem cell markers; and it is discovered that cells undergoing EMT acquire stem cell-like characteristics (stemness) showing a link between EMT and stem cell pathways, suggesting stemness, immune state, and EMT of cancer cells are closely related to each other in colorectal cancer 14. Stemness and EMT of CRC cells have been proved to promote tumor invasion and metastasis; and TGF-β/Smads pathway is indicated to promote stemness and EMT in CRC cells 15, 16. Therefore, stemness and EMT intervention in clinic are regarded as the potential targets in the CRC therapy.

**Figure 1:**
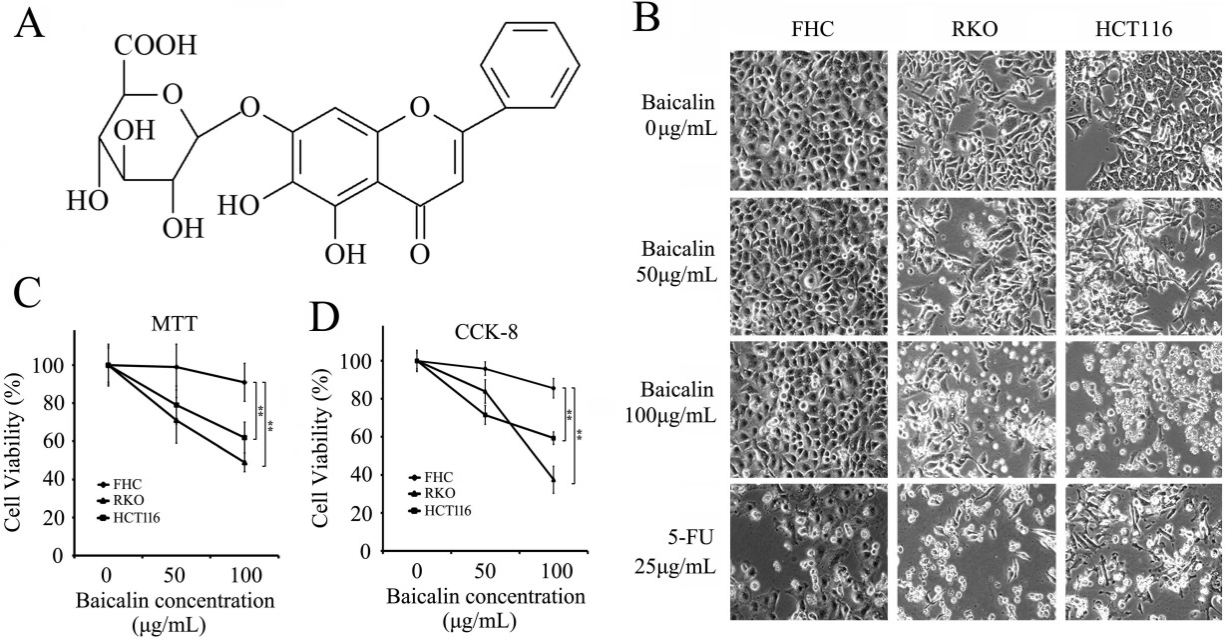
Effect of Baicalin on cell morphology and cell viability of colorectal normal mucosal cells and cancer cells. **(A)** Molecular structure of baicalin. **(B)** FHC, RKO and HCT116 cells were treated with various concentrations (0, 50 and 100 μg/mL) of baicalin and 5-FU (25 μg/mL) for 48 h and representative photographs of cell morphology were shown. **(C and D)** Cell viability after treatment with different concentrations of baicalin were checked by MTT (C) and CCK-8 assays (D). **p < 0.01.

Baicalin (7-D-glucuronic acid-5, 6-dihydroxyflavone; C_21_H_18_O_11_; molecular structure shown as Fig.1A) is a major active compound derived from the root of *Scutellaria baicalensis Georgi*, which is a traditional Chinese herb 17. It's reported that baicalin possesses obviously pharmacological effects including anti-cancer 18, 19, anti-inflammatory 20, anti-oxidant 21, 22 and neuroprotection 23. Recent Studies show that baicalin achieves its anti-cancer function by up-regulating the expression of Bax, Fas, FasL and Caspase-8, and down-regulating the expression of Bcl-2 in cervical cancer HeLa cells 24. In human leukemia cells, baicalin induces cell apoptosis via Bcl-2-dependent pathway 25. Also, baicalin can significantly inhibit PDGF-induced RASM cell proliferation and migration by suppressing the MAPK pathway 26.

Here, *in vitro* and *in vivo* experimental data showed that baicalin prevents cell cycle progress by blocking cell transition from G1 phase to S phase (arresting cell cycle at G1-phase), inhibits EMT of CRC cells by inhibiting TGFβ/Smads pathway, and attenuates CRC cell stemness by decreasing the levels of cancer stem cell markers; and simultaneously, baicalin also initiates and induces CRC cell apoptosis by activating Caspase-dependent signal pathways. Finally, baicalin treatment in CRC cells induces cell growth inhibition and apoptosis, suggesting it may be a great candidate in treating patients with colorectal cancer in clinic by comprehensively targeting and suppressing cell cycle, EMT and stemness of CRC cells.

## Materials and Methods

### Cell culture and stem cell-like sphere formation

Human colorectal cells lines (including FHC, RKO and HCT116) were purchased from American Type Culture Collection (Manassas, VA, USA) and cultured in RPMI-1640, Eagle's Minimum Essential Medium and McCoy's 5A, respectively. All mediums were supplemented with 10% fetal bovine serum (FBS, BRL-GIBCO Co. Ltd., CA, USA), 100 mg/ml streptomycin and 100 U/ml penicillin. Cells were placed in the incubator with 37°C, 5% CO_2_ air atmosphere.

For the formation of stem cell-like spheres, HCT116 cells were suspended in serum-free McCoy's 5A medium containing B27 (1:50, BRL-GIBCO Co. Ltd., CA, USA), recombinant human epidermal growth factor (rhEGF, 20 ng/mL, PeproTech, NJ, USA) and recombinant human fibroblastic growth factor-basic (rhFGF-b, 20 ng/mL, PeproTech) in ultralow-attachment 6-well plates (Corning, Switzerland). For subculture and reformation of cell spheres, the preformed cell spheres were collected by centrifugation, trypsinized (to single cell), counted and replanted in new McCoy's 5A medium in ultralow-attachment 6-well plates.

### Reagents and antibodies

Baicalin with 98% purity was purchased from National Institute for the Control of Pharmaceutical and Biological Product (Hangzhou, China); 5-FU was obtained from Yuanye Biological Technology (Shanghai, China). Baicalin and 5-FU were dissolved in dimethyl sulfoxide (DMSO). TGF-β1 was purchased from PeproTech and treated cells for 12 h in this study. Antibodies for Smad3, p-Smad3, Smad2, p-Smad2 and Smad4 were purchased from Cell Signaling Technology (Boston, MA, USA). Antibodies for Smad7, Akt, p-Akt, Cyclin B1, Cyclin D1, P21, P53, Parp-1, Caspase 3, XIAP, Survivin and β-actin were purchased from Santa Cruz Biotechnology Inc. (Santa Cruz, CA. USA). Antibodies for CD133, CD44, Nanog, OCT4, SOX2, Bcl-2, Bax, P27, Caspase8, Caspase9, Snail, Twist and Slug were obtained from Proteintech (Rosemont, IL, USA). Antibodies for TGF-β1, N-Cadherin, E-Cadherin, Vimentin, Cytokeratin 18, Claudin 1, NF-κB-p65 and Cyclin E1 were purchased from Bioworld Technology Inc. (St Louis, MN, USA).

### MTT assay and CCK-8 assay for cell viability

MTT assay and CCK-8 (Cell Counting Kit-8) assay were performed to check the cell viability. Cells were seeded in a 96-well plate at a density of 2×10^4^ cells/well overnight and treated with different concentrations of baicalin as indicated in figures. For MTT assay, culture medium was removed and fresh medium (100 μL) was added with 10 μL of MTT (5 mg/mL). The plate was incubated at 37°C for 4 h in the dark. The medium was removed again, and 100 μL of DMSO was added to each well. The absorbance at 570 nm was measured by a microplate reader (Thermo Scientific, Fremont, CA, USA). For CCK-8 assay, culture medium was removed and fresh medium (100 μL) was added with CCK-8 solution (5 μL). The plate was incubated at 37°C for 4 h in the dark. Absorbance at 450 nm was measured by a microplate reader. The measured OD values were converted into cell viability according to the manufacturer's protocol.

### DAPI staining assay for cell apoptosis

For DAPI staining assay, FHC, RKO and HCT116 cells were cultured in 12-well plates and incubated with different concentrations of baicalin as indicated in figures (25 μg/ml of 5-FU as positive control). After 48 h, cells were washed with 1× PBS briefly and fixed in 4% formaldehyde for 15 min, and washed three times with 1×PBS and then permeabilized in 0.2% Triton X-100 for 15 min. Cells were then stained with DAPI (20 μg/mL in 1×PBS) at room temperature for 8 min and finally were photographed by fluorescence microscopy (Nikon, IX-71, Japan).

### Western-blot assay and flow cytometry analysis

The total proteins were extracted from the harvested cells by RIPA Lysis Buffer (Beyotime, China) with protease inhibitor cocktail according to the manufacturer's instructions. Equal amounts of total protein were separated by SDS-PAGE and western-blot assay. The target proteins were detected by specific antibodies and Enhanced Chemiluminescence Detection Kit (Amersham Bioscience, Pittsburgh, PA, USA). The protein bands were quantified using the Analysis software with Bio-Rad gel imager (β-actin as the internal reference); and then the synthetic results (values) were gotten and presented under the western blot bands in figures.

For flow cytometry analysis, cells treated with different concentrations of baicalin were harvest, washed with PBS, and then stained by using an Annexin V-FITC/PI Apoptosis Detection Kit (KeyGen Biotech, Nanjing, China) according to the manufacturer's instructions. Cellular apoptosis assay was performed on a flow cytometer (BD Accuri™ C6 flow cytometer, BD Biosciences). For cell cycle assay, cells were harvested, washed with PBS, fixed and stained by using Cell Cycle Detection Kit (KeyGen Biotech, Nanjing, China) according to the manufacturer's instructions. Cell cycle distribution was measured by flow cytometer analysis.

### Cell adhesion and colony formation assays

Wells of fibronectin (FN)-coated 96-well plate were pre-washed (2 times) and pre-blocked (37°C for 1 h) with the washing/blocking buffer (containing 0.1% BSA). 2,000 cells/well were seeded into pretreated 96-well plate and incubated at 37°C for 2 h. After washing with PBS, cells were fixed with 4% paraformaldehyde and stained with staining buffer containing 0.1% crystal violet. Then cells were washed with PBS and photographed with microscope (Nikon microscope, Japan). After photographing, cells were added 2% SDS and incubated at room temperature (RT) for 30 min. The absorbance of each well at 570 nm was measured by a microplate reader and the adherence inhibition rates were calculated.

For colony formation assay, 500 cells/well were seeded into a 12-well plate and incubated for 24 h, and then treated with different concentrations of baicalin for 4 days or 7 days as indicated in figures. The cells were washed twice with PBS and stained with 0.1% crystal violet solution; and then the colonies were observed and photographed under an inverted microscope.

### Cell migration and invasion assays

For wound healing assay, cells were seeded in 6-well plates in complete culture medium and cultured to 80% confluent. The cell layer was carefully wounded using a sterile tip and washed twice with fresh serum free media, and then cultured in medium with 0.5% FBS and treated with different concentration of baicalin as indicated in figures. Photographs of the same wound area were taken at different time points (as shown in figures) to determine the migration ability of cells treated with different concentration of baicalin.

In addition, cell migration and invasion assays were performed by using transwell chambers (pore size of 8 µm; Costar, Corning, Switzerland), which bottom was coated with 1 mg/ml BD Matrigel Matrix (BD Biosciences, USA) for invasion assay specifically. The inserts (transwell chambers) were placed in the 24-well culture plates to form upper and lower chambers. 200 μL/well serum-free medium containing 1×10^5^ cells and different concentration of baicalin were added to upper chambers with uncoated (for migration assays) or Matrigel coated (for invasion assays) bottom. Meantime, 500 µL/well medium containing 20% FBS were added to lower chambers. After incubating 48h, the non-invasive/non-migration cells were removed by cotton swabs, and the invaded/ migrated cells, which stucked to the lower surface of the filters, were fixed with cold 100 % methanol for 30 min and then stained with 0.1 % crystal violet solution for 30 min. Finally, the invaded/migrated cells were washed with PBS and photographed under a microscope with digital imaging system (Olympus DP50, Olympus, Japan) in the appropriate magnification.

### Construction of orthotopic transplanted colon tumor models in nude mice and anti-tumor assay of baicalin* in vivo*

This study on experimental animals was approved (Permission No: NL-129-02) by the Ethics Committee of Jiangsu Province Hospital of TCM, Nanjing, China. BALB/c nude mice (6 weeks) were purchased from Model Animal Research Center of Nanjing University, Nanjing, China; and all operations were based on the guidelines of Committee on Animals of the Chinese Academy of Sciences. The stable GFP expression HCT116 cells (HCT116-GFP) were constructed by our lab, and then HCT116-GFP cells were subcutaneously injected into 6-week-old nude mice. When tumor size was ~500 mm3, the tumors were separated from HCT116-GFP xenograft mice and cut into small particles of the same size (almost 1 mm3/each); and then one particle of each tumor was transplanted into the colonic epithelium of mice in situ by surgery.

After the orthotopically transplanted colon tumors grew to 50-100 mm3, mice in half genders were randomly grouped and treated with diluted DMSO (0.5mL DMSO diluted with 13.5mL NS; as negative control, NC), 100 mg/kg of baicalin (low dose group, LD), 200 mg/kg baicalin (high dose group, HD) and 25 mg/kg of 5-FU (as positive control, PC), respectively. Mice were treated by intragastrical administration every day for four weeks (8 mice/group for tumor growth inhibition assay, 8 mice/group for tumor metastasis inhibition assay and 7 mice/group for survival assay) with negative/ positive control reagents and low-/high- doses of baicalin. For tumor growth inhibition assay, the tumor size and mice weight were measured at 1, 4, 8, 11, 15, 18, 22, 26, 29 and 32 days; and after tumor growth for 32 days, the mice were sacrificed and the orthotopically transplanted tumors were photographed *in vivo*; and then the tumors were isolated, weighted and equally dissected into two parts. One part of tumors was frozen in liquid nitrogen and stored at -80°C for western-blot assay, and another part was formalin fixed and paraffin embedded for immunohistochemistry (IHC). For tumor metastasis inhibition assay, mice were raised to two months after treated with baicalin and controls, and then sacrificed for checking tumor metastasis into different tissues with a fluorescence stereo microscope (MZ650; Nanjing Optic Instrument Inc., China), and tumor volume was calculated using Image-Pro plus 6.0 software (Media Cybernetics, Silver Spring, MD, USA) based on the length (L) and width (W) of the tumor. For survival survey, mice of different groups were raised to death and the days of mice lifetime were recorded; and then the Kaplan Meier curves were made using SPSS software.

### Immunohistochemistry (IHC) analysis

Tumor tissues were fixed in 10% buffered formalin, embedded in paraffin, sectioned in 5 μm size. Each tissue section was deparaffinized and rehydrated with upgraded ethanol; and then tissue sections were boiled in EDTA for 15 min, quenched with 0.3% hydrogen peroxide solution for 10 min at room temperature and blocked with BSA in PBS for 30 min. Slides were subsequently incubated with special primary antibodies as indicated in figures overnight at 4°C. Sections were counterstained with hematoxylin. Antibody binding was detected with an Envision Detection Kit, Peroxidase/DAB, Rabbit/Mouse (Gene Tech, Shanghai, China). The expression levels of special proteins (as indicated in figures) were observed and photographed under a microscope at a magnification of 400× (CTR 6000; Leica, Wetzlar, Germany).

### Statistical analysis

All data were analyzed with SPSS 17.0 statistics software and shown as mean ± standard error of the mean (S.E.M). Comparison between two groups was analyzed using the unpaired Student's t-test and comparison between more than two groups was analyzed by a one-way ANOVA analysis. Differences were considered statistically significant at *p < 0.05, **p < 0.01 and ***p < 0.001.

## Results

### Baicalin decreased cell viability and induced apoptosis in CRC cells

Normal colonic epithelial cells (FHC) and colorectal cancer cells (RKO and HCT116) were cultured in 96-well plates and treated with increasing doses of baicalin as indicated in Figure 1 (1B, 1C and 1D) for 48 h (5-FU, as positive control). From the data, we found that floating cells, dead cells and cell debris in the cultures of RKO and HCT116 cells were much more than those of FHC cells and increased with the increasing treated concentration of baicalin; and baicalin treatment also thinned the adherent cells in two cancer cell lines (Fig. 1B). Baicalin has little effect on FHC cells (Fig. 1B). MTT and CCK-8 assays showed that 100 μg/mL of baicalin significantly suppressed the cell viabilities of RKO and HCT116 respectively, while the cell viability of FHC was not significantly changed (Fig. 1C and 1D).

**Figure 2:**
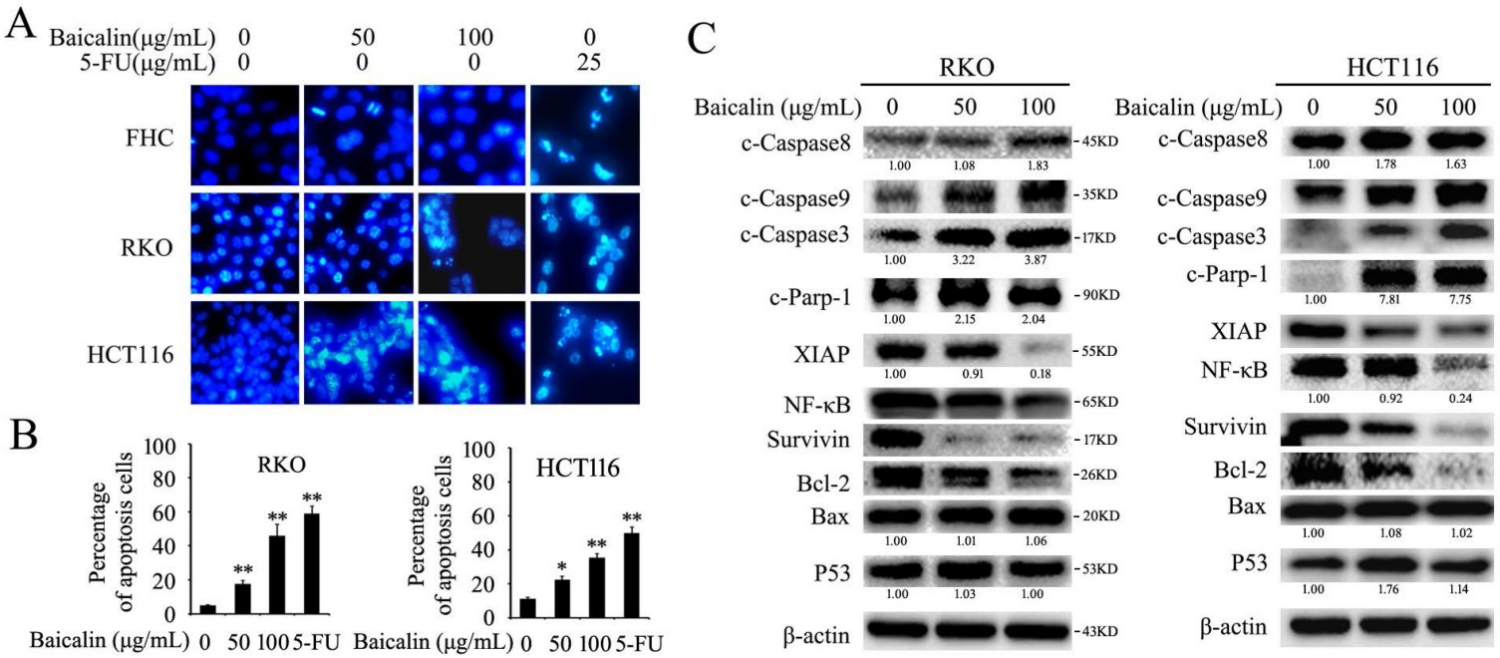
Baicalin treatment induced apoptosis in CRC cells via regulating apoptosis-related proteins. **(A)** FHC, RKO and HCT116 cells were treated with different concentrations of baicalin (0, 50 and 100 μg/mL) and 5-FU (25 μg/mL) for 48 h, and then cells were stained with DAPI and analyzed by fluorescence microscopy. **(B)** Cells which treated with 0, 50 and 100 μg/mL of baicalin and 25 μg/mL of 5-FU for 48 h were assayed for apoptosis by flow cytometry with Anexin V / propidium iodide double-staining. The percentage of apoptotic cells (including early and late apoptotic cells) in total cells were quantified and showed. *p < 0.05, **p < 0.01. **(C)** RKO and HCT116 cells were treated with different concentrations of baicalin (0, 50 and 100 μg/mL) for 48 h. The levels of apoptosis-related proteins (cleaved caspase-3, -8, -9, cleaved Parp-1, XIAP, NF-κB, Survivin, Bcl-2, Bax and p53) and β-actin (an internal control) were analyzed by western blot analysis. The relative protein levels in western-blot assay were quantified and marked under the protein bands.

DAPI staining assay showed that number of cells with fragmented nuclei was increased with the increasing concentration of baicalin in both RKO and HCT116 cells, while a weak effect of baicalin on FHC cells was observed (Fig. 2A). Furthermore, annexin V/propidium iodide (AV/PI) staining and Flow cytometry assays were also showed that baicalin treatment greatly induced apoptosis of RKO and HCT116 cells in a dose-dependent manner. Specially, when treated with 100 μg/mL of baicalin, the percentages of total apoptotic cells (including early and late apoptotic cells) of RKO and HCT116 were up to 46.8% and 35.7% respectively (Fig. 2B and Fig. S1).

Western-blot data showed that baicalin treatment increased the protein levels of cleaved caspase 3/8/9 (c-Caspase 3, c-Caspase 8 and c-Caspase 9) and cleaved PARP-1 (c-PARP-1) and decreased the protein levels of XIAP, NF-κB, Survivin and Bcl-2 in a dose-dependent manner in both RKO and HCT116 cells, whereas the protein levels of Bax and P53 were not changed obviously (Fig. 2C).

These results indicated that baicalin could reduce cell viability and induce caspase/PARP-1 dependent apoptosis in CRC cells.

### Baicalin inhibited cell proliferation and growth by suppressing colony formation and cell adhesion in CRC cells

**Figure 3:**
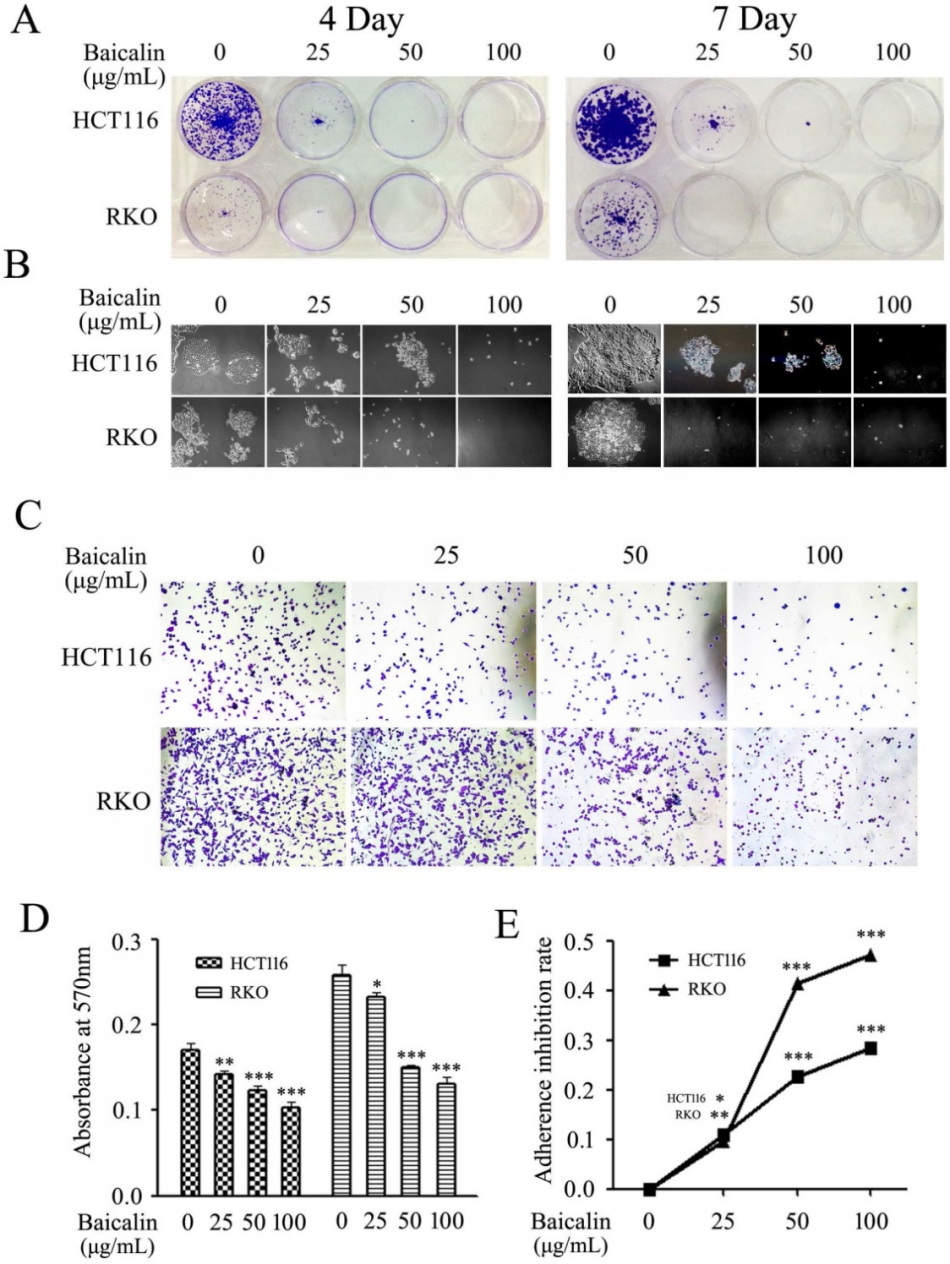
Baicalin treatment inhibited cell growth and decreased cell adhesion ability in CRC cells. **(A)** RKO and HCT116 cells treated with 0, 25, 50 and 100 μg/mL of baicalin were incubated for 4 days or 7 days, respectively; and then the colonies were stained and photographed with microscope under 10 × magnification. **(B)** The representative colonies were captured with microscope under 40 × magnifications. **(C)** RKO and HCT116 cells were cultured with the medium containing 0, 25, 50 and 100 μg/mL of baicalin in an incubator for 2 h and then adherent cells were stained and photographed with microscope. **(D and E)** The stained adherent cells were treated with 2% SDS buffer and the cell adhesion abilities were quantified by measuring the absorbance at 570 nm with a microplate reader and the adherence inhibition rates of cells were calculated. *p < 0.05, **p < 0.01, ***p < 0.001.

Colony formation and cell adhesion assays were employed to evaluate the effect of baicalin on the proliferation and growth in CRC cells. From our experimental data, baicalin treatment greatly decreased the number and size of formed colonies in RKO and HCT116 cells by comparing with those of untreated cells; and the inhibition degree was dependent on the treated concentration and treated time of baicalin (Fig. 3A and 3B).

Results of cell adhesion assay showed that the adhesion abilities of CRC cells on fibronectin (FN)-coated surface were significantly reduced by the treated baicalin in a concentration dependent manner in both RKO and HCT116 cells (Fig. 3C). Quantitative analysis also demonstrated that adhesion cells were remarkably decreased with the treatment of baicalin in a concentration-dependent manner in two cell lines (Fig. 3D and 3E).

These results elucidated that baicalin treatment in colorectal cancer cells inhibited cell colony formation and suppressed the adhesion of cancer cells to FN, indicating that baicalin inhibited the proliferation and growth of colorectal cancer cells.

### Baicalin inhibited cell growth by arresting the cell cycle mainly at G1-phase in CRC cells

**Figure 4:**
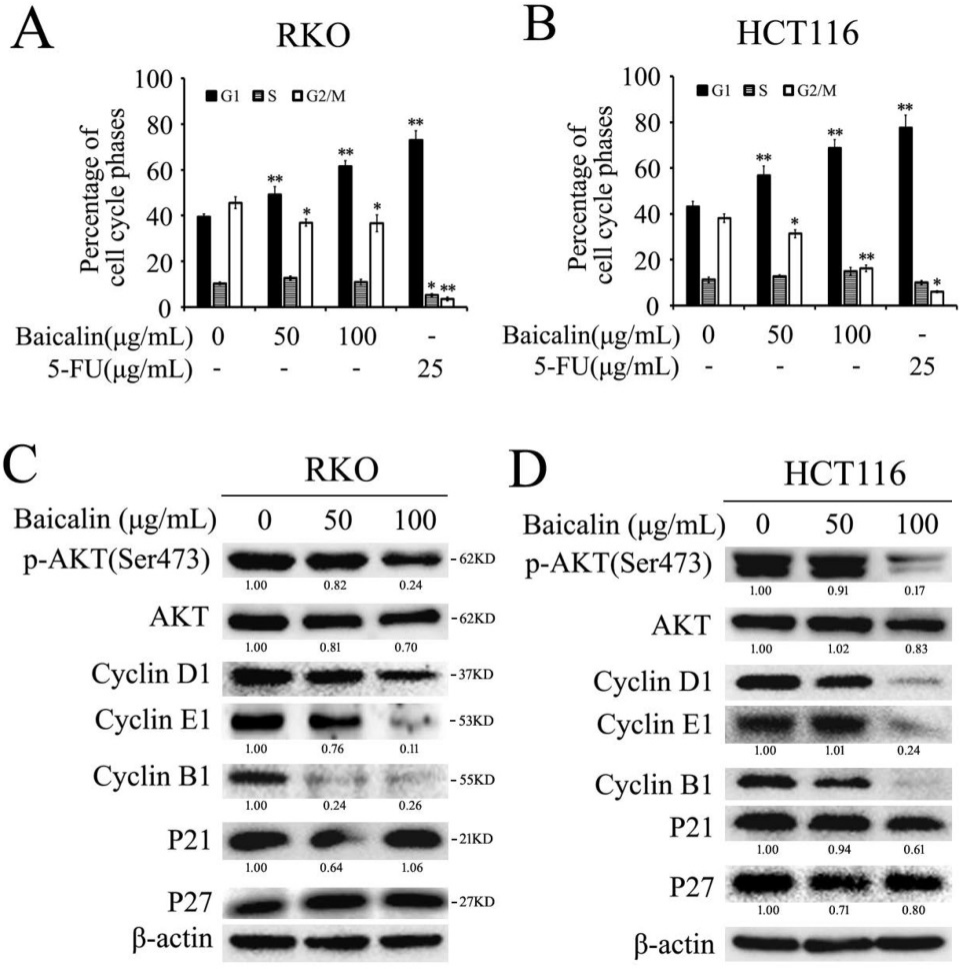
Baicalin treatment induced cell cycle arrest mainly in G1 phase in RKO and HCT116 cells. **(A and B)** RKO and HCT116 cells were treated with different concentrations of baicalin (as indicated in figures) and 5-FU for 48 h; and then cells were stained with propidium iodide (PI) and cell cycle distributions were analyzed by flow cytometric analysis. The percentages of cells in different phases were calculated and presented. *p < 0.05, **p < 0.01.** (C and D)** Cells were treated as above and harvested, and then the protein levels of p-Akt, Akt, Cyclin D1, Cyclin E1, Cyclin B1, P21, P27 and β-Actin were analyzed by western blot analysis. The relative protein levels in western-blot assay were quantified and marked under the protein bands.

Flow cytometry assay was employed to investigate the effect of baicalin treatment on cell cycle progression in CRC cells. From the results, we found that baicalin treatment in RKO and HCT116 cells could induce cell arrest in G1-phase; and the accumulation of cells in G1-phase was dependent on the treated concentration of baicalin (Fig. 4A and 4B). Correspondingly, baicalin could distinctly reduce the percentage of G2/M phase cells, but the percentage of S phase cells did not change significantly (Fig. 4A and 4B).

To further investigate the major underlying mechanisms of baicalin-induced cell cycle arrest, we checked the effect of baicalin on the cell cycle-related proteins. From the western blot data, baicalin treatment dramatically decreased the levels of Cyclin D1, Cyclin E1, Cyclin B1 and p-Akt (Ser473) in all two CRC cell lines. Moreover, we found that baicalin had little impact on the levels of p21 and p27 (Fig. 4C and 4D).

These results indicated that baicalin-induced cell cycle arrest was due to a direct decrease in levels of cyclins associated with CRC cell cycle, rather than activation of p53/p21/p27 pathway.

### Baicalin treatment inhibited cell migration and invasion in CRC cells

**Figure 5:**
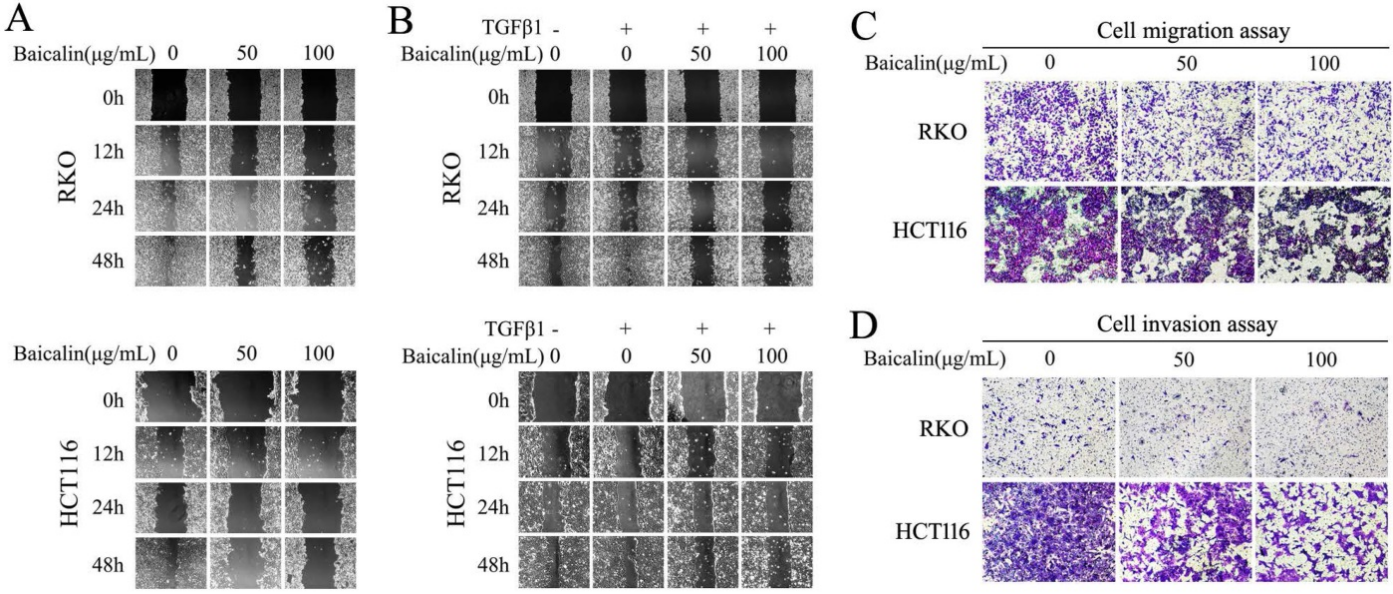
Baicalin treatment inhibited cell migration and invasion in CRC cells. **(A)** RKO and HCT116 cells were cultured and treated with different concentrations of baicalin as indicated in figures, and wound healing assay was carried out and the gaps were photographed at 0 h, 12 h, 24 h and 48 h by using an invert microscope, respectively. **(B)** RKO and HCT116 cells were starved with 0.5% FBS for 12 h and then treated with/without TGF-β1 and different concentrations of baicalin as indicated in figures. Then the gaps were photographed as above. **(C and D)** After starvation with 0.5% FBS for 12 h, RKO and HCT116 cells were treated with different concentrations of baicalin as indicated in figures. The cell motility, migration and invasion were assayed by transwell assays and the migrated and invaded cells were stained and photographed.

To identify the potential role of baicalin in inhibiting the migration and invasion of CRC cells, wound-healing assay and transwell assay were carried out. From our experimental data, wound-healing assay showed that baicalin treatment in RKO and HCT116 cells had a slow closure of the scratch wound gaps by comparing with untreated cells. The migratory distances of CRC cells with baicalin treatment were smaller than those of untreated cells (Fig. 5A). Furthermore, TGFβ1-induced cell migration was also impaired by treated baicalin (Fig. 5B).

The transwell experimental data, including transwell chambers with and without Matrigel, showed that baicalin treatment dramatically decreased the number of cells migrating (Fig. 5C) or invading (Fig. 5D) to the lower chamber in a concentration dependent manner. These results demonstrated that baicalin could effectively prevent the migration and invasion of colorectal cancer cells.

### Baicalin inhibited EMT of CRC cells by targeting TGFβ1/Smad pathway

**Figure 6:**
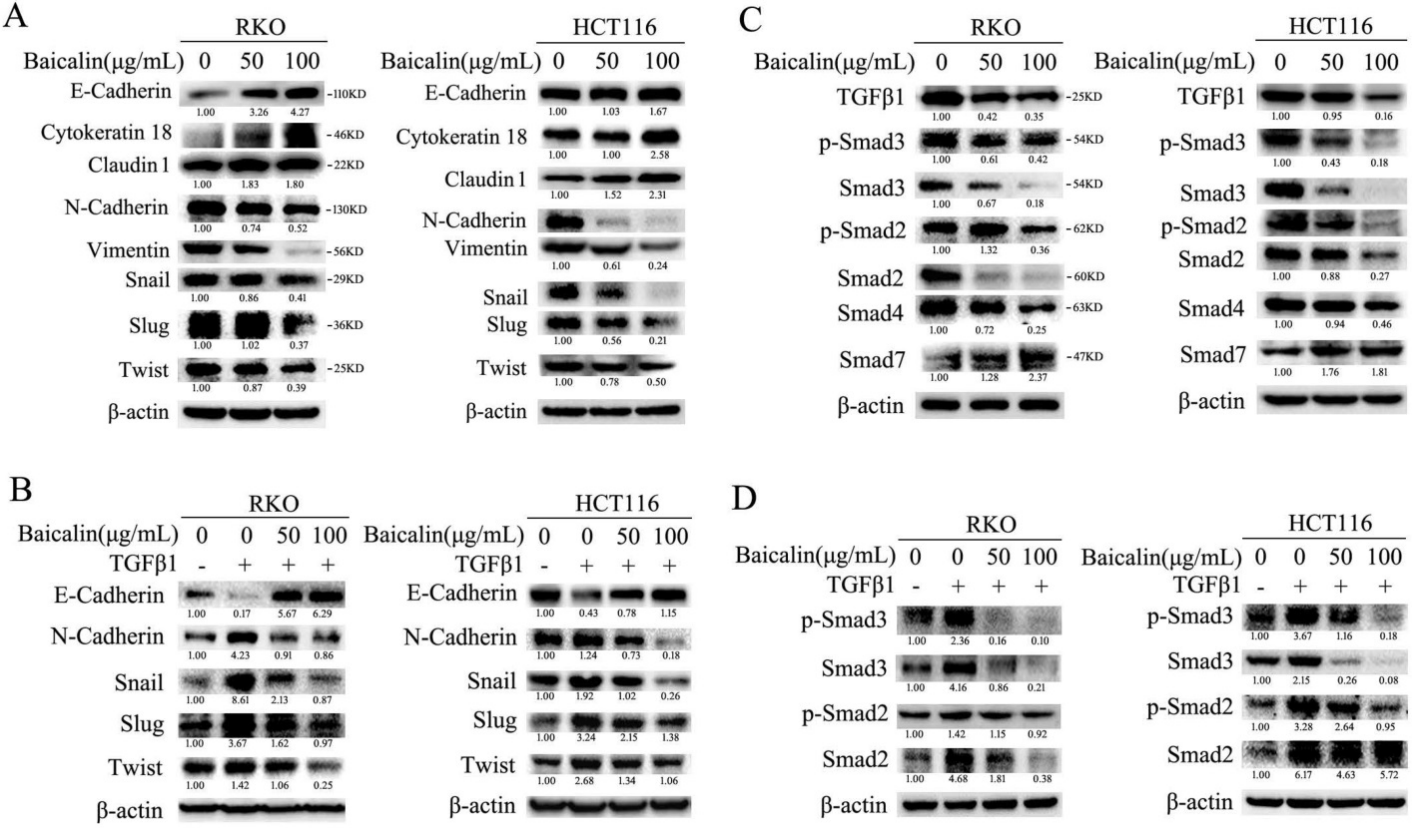
Baicalin inhibited epithelial to mesenchymal transition (EMT) by suppressing TGF-β/Smad pathway in CRC cells. **(A)** RKO and HCT116 cells were treated with different concentrations of baicalin for 48 h, and then cells were harvested and the protein levels of E-cadherin, Cytokeratin 18, Claudin 1, N-Cadherin, Vimentin, Snail, Slug, Twist and β-actin were determined by western blotting. **(B)** After treating with different concentrations of baicalin for 48 h, RKO and HCT116 cells were treated or untreated with TGF-β1 (10 ng/mL) for another 12 h; and then cells were harvested. The protein levels, including E-cadherin, N-cadherin, Snail, Slug and Twist, were detected by western blot analysis. **(C)** RKO and HCT116 cells were incubated with increasing doses of baicalin and total cell lysates were analyzed by immunoblotting with anti-phospho Smad2/3, anti-Smad2/3/4/7 antibody. **(D)** After treating with different concentrations of baicalin for 48 h, RKO and HCT116 cells were treated or untreated with TGF-β1 (10ng/mL) for another 12 h; and then cells were harvested. The protein levels of p-Smad2/3 and Smad2/3 were analyzed by immunoblotting. The relative protein levels in western-blot assay were quantified and marked under the protein bands.

To investigate the effect of baicalin on the EMT (epithelial to mesenchymal transition) of CRC cells, the levels of proteins associated with cell EMT were analyzed by immunoblotting. The experimental data showed that baicalin treatment in CRC cells increased the protein levels of cell epithelial markers, such as E-cadherin, Cytokeratin 18 and Claudin1, while decreased the protein levels of cell mesenchymal markers, such as N-cadherin and Vimentin (Fig. 6A). Moreover, the protein levels of EMT-associated transcription factors, such as Snail, Slug and Twist, were also down-regulated (Fig. 6A). In addition, we found that baicalin treatment could up-regulate exogenous TGFβ1-reduced E-cadherin and down- regulate exogenous TGFβ1-enhanced N- cadherin, Snail, Slug and Twist in CRC cells (Fig. 6B). All the above changes of baicalin- induced protein levels were dose- dependent (Fig. 6A, 6B).

Next, the effect of baicalin on TGFβ/ Smad pathway-related proteins was investigated. Surprisingly, the experimental data showed that baicalin treatment in CRC cells decreased the protein levels of endogenous TGFβ1, p-Smad2/3, Smad2/3 and Smad4, while increased the protein level of Smad7 (Fig. 6C). Furthermore, baicalin treatment also decreased exogenous TGFβ1-induced up-regulation of p-Smad2/3 and Smad2/3 in CRC cells (Fig. 6D). All the above baicalin-induced protein changes were dose-dependent (Fig. 6C, 6D).

These results identified that baicalin could inhibit cell EMT by suppressing TGFβ/Smad signaling pathway in CRC cells.

### Baicalin suppressed the stemness of cells and inhibited the formation of stem cell-like spheres in CRC cells

**Figure 7:**
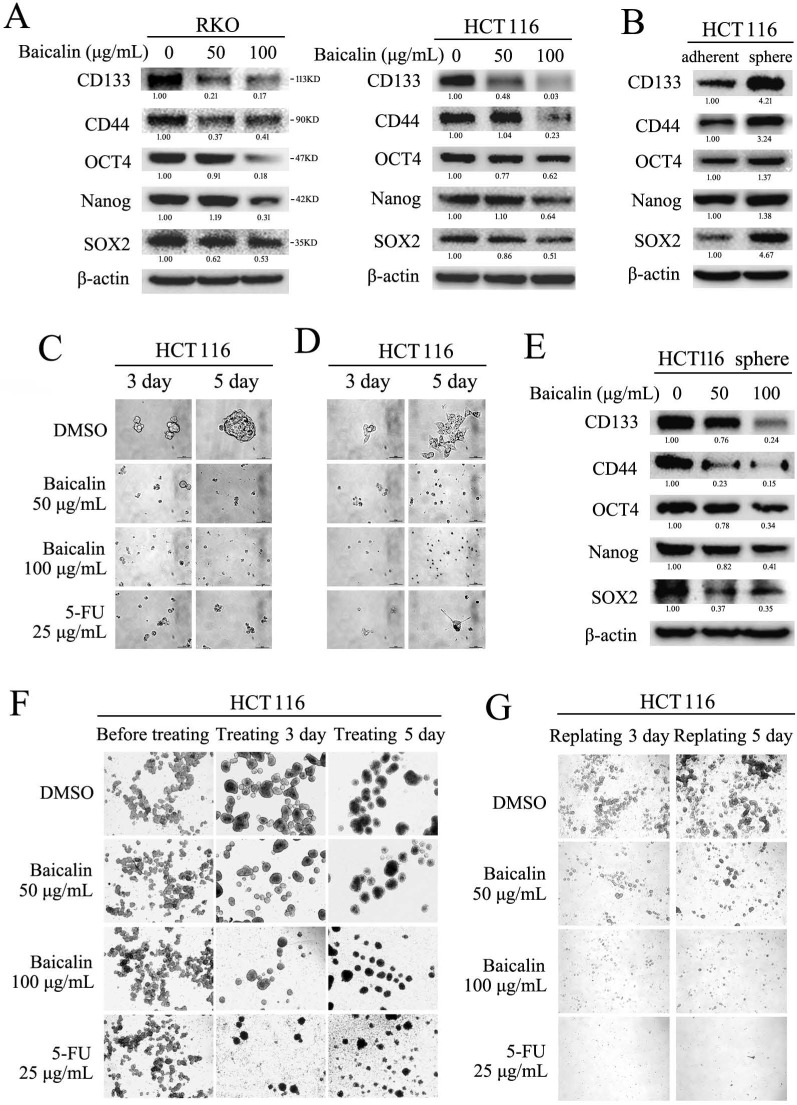
Baicalin inhibited formation of stem-like cell spheres and decreased stemness markers in CRC cells. **(A)** RKO and HCT116 cells were treated with different concentrations of baicalin (as indicated in figures) for 48 h, and protein levels of cell stemness markers, including CD133, CD44, SOX2, OCT4 and Nanog, were checked by western-blot assay. **(B)** Protein levels of the stemness markers in both adherent-cultured and sphere-cultured HCT116 cells were checked by western-blot assay. **(C)** HCT116 cells were seeded in ultralow-attachment 6-well plates and cultured in the medium containing different concentrations of baicalin (0, 50 and 100 μg/mL) for 3 days and 5 days (5-FU as positive control); and the formed stem-like HCT116 cell spheres were photographed. **(D)** The pre-formed HCT116 cell sphere was trypsinized into single cells, and then the same number of cells were adherently cultured in normal plates with the medium containing different concentrations of baicalin (0, 50 and 100 μg/mL; 5-FU as positive control); and the cell growth were photographed at 3 days and 5 days. **(E)** The effect of baicalin on the protein levels of stemness markers in the formed HCT116 cell spheres (at 48 h) were analyzed by western-blot assay. **(F)** The pre-formed HCT116 cell spheres (one week) were treated with different concentrations of baicalin (0, 50 and 100 μg/mL) for 3 days and 5 days (5-FU as a positive control), and then the HCT116 cell spheres were photographed. **(G)** The formed HCT116 cell spheres were trypsinized into single cell and then replanted in ultralow-attachment 6-well plates again with the medium containing different concentrations of baicalin and 5-FU (as positive control) for 3 days and 5 days. Propagation and reformation of HCT116 cell spheres were photographed. The relative protein levels in western-blot assay were quantified and marked under the protein bands.

Stem cell-like characteristics (stemness) of cancer cells played critical roles in tumor metastasis, drug-resistance and tumor recurrence in clinic 27, 28. To assay the effect of baicalin on the stemness of CRC cells, RKO and HCT116 cells were cultured and treated with different concentration of baicalin. Western-blot data showed that baicalin treatment obviously decreased the protein levels of stem cell markers (including CD133, CD44, SOX2, OCT4 and Nanog) in a concentration- dependent manner in both RKO and HCT116 cells (Fig. 7A).

Formation assay of stem cell-like spheres (in ultralow-attachment plate) was employed to further evaluate the effect of baicalin on the stemness of CRC cells. The experimental data showed that the protein levels (including CD133, CD44, SOX2, OCT4 and Nanog) in HCT116 cell spheres were increased by compared with adherent cultured HCT116 cells (Fig. 7B). Baicalin treatment greatly inhibited the formation of HCT116 cell spheres in a dose-dependent manner (Fig. 7C). Western-blot data showed that baicalin decreased the protein levels of CD133, CD44, SOX2, OCT4 and Nanog in a dose-dependent manner in sphere-cultured HCT116 cells (Fig. 7E). Further, baicalin treatment could also inhibit cell growth when the sphere-cultured HCT116 cells were adherently cultured again in normal plate (Fig. 7D). In addition, we treated pre-formed HCT116 cell spheres (formation for one week) with different concentration of baicalin to check the effect of baicalin on the growth of formed HCT116 cell spheres. The data showed that baicalin treatment promoted the fusion of HCT116 cell spheres and inhibited the growth of pre-formed HCT116 cell spheres in a concentration dependent manner (Fig. 7F). Similarly, baicalin treatment inhibited propagation and reformation of HCT116 cell spheres in a concentration dependent manner (Fig. 7G). These results demonstrated that baicalin treatment could decrease the stemness-related protein levels and inhibit the formation and growth of stem cell-like spheres in CRC cells.

### Baicalin suppressed tumor growth and induced apoptosis of CRC cells in orthotopic xenograft model

**Figure 8:**
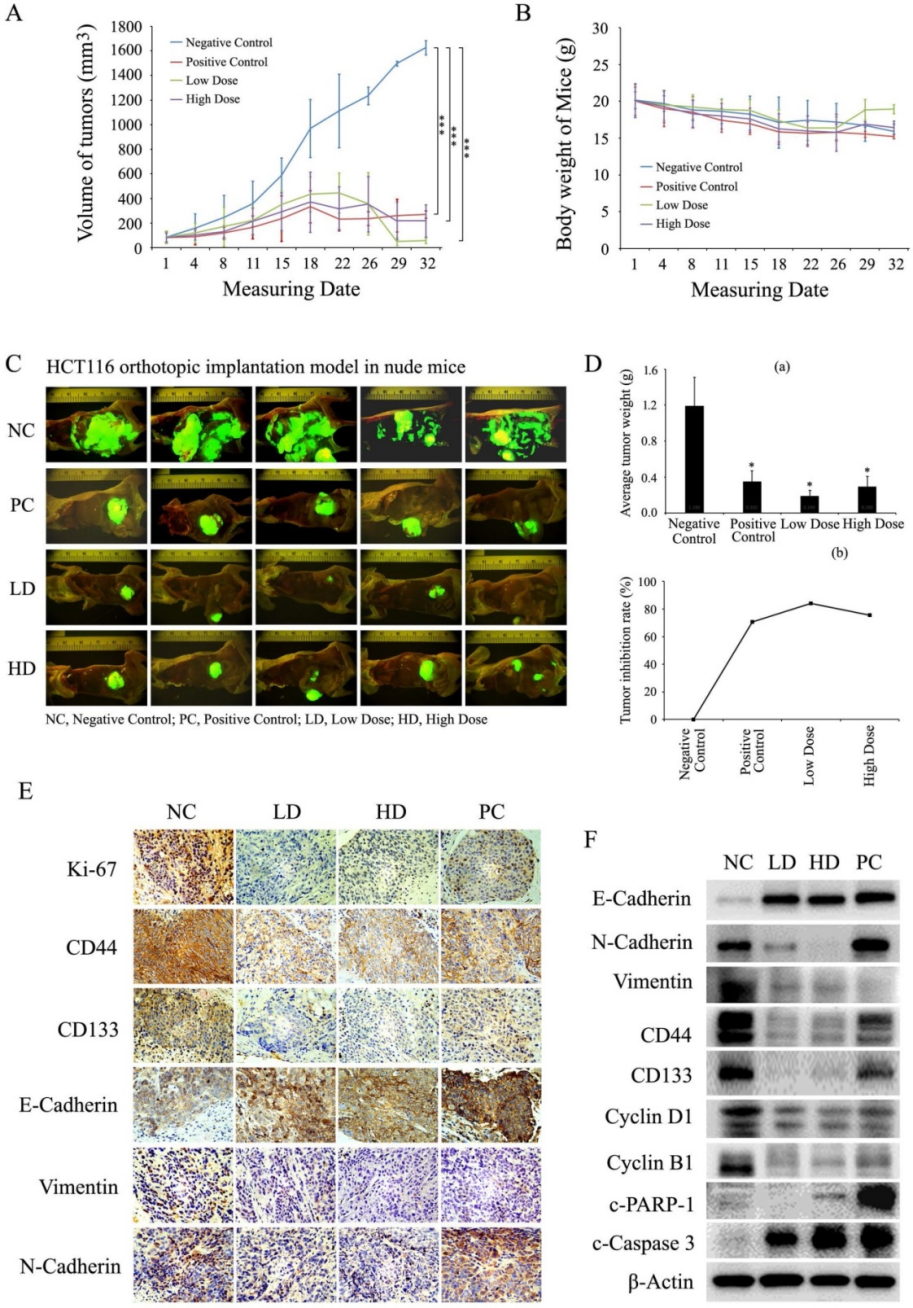
Baicalin inhibited tumor growth and induced apoptosis *in vivo* by decreasing cell growth-related proteins and increasing apoptosis-related proteins. **(A and B)** Mice with orthotopically transplanted colon tumors were randomly divided into four groups and given intragastric administration every day for four weeks with negative control (NC), positive control (PC), low-dose baicalin (LD) and high-dose baicalin (HD) as described in “Materials and Methods”. The tumor size and mice weight were measured at 1, 4, 8, 11, 15, 18, 22, 26, 29 and 32 days and then presented in line charts. ***p < 0.001.** (C)** After tumor growth for 32 days, the mice were sacrificed and the orthotopically transplanted tumors were photographed by Inverted Fluorescence Microscope. **(D)** Tumors of each treatment group were isolated and weighted and plotted (a), *p < 0.05; and the tumor growth inhibition rate (%) in each treatment group was calculated and plotted (b).** (E and F)** Tumor tissues were dissected into two parts, one part was fixed with formaldehyde and embedded by paraffin for IHC experiments by using the specific antibodies against Ki67, CD44, CD133, E-Cadherin, N-Cadherin and Vimentin, respectively; another part was lysed with lysis buffer for western-blot experiments to assay the levels of CD44, CD133, E-Cadherin, N-Cadherin, Vimentin, Cyclin D1, Cyclin B1, c-Caspase 3, c-PARP-1 and β-Actin (as internal control), respectively.

To investigate the effect of baicalin on CRC cells in vivo, HCT116 (with stable expression of GFP) orthotopic transplanted colorectal tumor models in nude mice were established to evaluate the inhibition of tumor growth by baicalin treatment. From the results, baicalin treatment *in vivo* obviously inhibited the growth of HCT116 orthotopic transplanted colon tumors, and yet there were no significant changes in body weights among high dose baicalin-treated group, positive control groups and negative control group (Fig. 8A and 8B). Interestingly, at the end of experiment, tumor-growth inhibition of low-dose group was a little more significant than those of high-dose and positive control groups by compared with that of negative control group (Fig. 8A, 8C and 8D (a)); and the mice body weight of low-dose group was a little heavier than those of other three groups (Fig. 8B). In addition, the final tumor inhibition rates (ratios of average tumor size of treated groups to that of negative control group) also showed that the tumor inhibition efficacy of baicalin in low-dose group were better than those in both high-dose group and positive control group (Fig. 8D (b)).

Furthermore, data of immunohistochemical analysis demonstrated that baicalin treatment *in vivo* enhanced the expression of E-cadherin and reduced the expressions of Ki67, Vimentin, N-cadherin, CD133 and CD44 by comparing with negative control in HCT116 orthotopic transplanted colorectal tumors (Fig. 8E). Western blot assay showed that baicalin treatment up-regulated the protein levels of E-cadherin, c-PARP-1 and c-Caspase 3 and down-regulated the protein levels of Ki67, Cyclin D1, Cyclin B1, Vimentin, N-cadherin, CD133 and CD44 by comparing with those of negative control (Fig. 8F). In addition, the protein levels of N-cadherin, CD44 and CD133 in positive group were higher than those in baicalin-treated groups (Fig. 8F).

These results demonstrated that baicalin treatment could inhibit the growth of orthotopic transplanted colon tumors in nude mice by down-regulating the levels of stemness-related, EMT-related and cell cycle-related proteins and up-regulating the levels of apoptosis-related proteins. Low dose baicalin (compared with high dose baicalin) had a little higher inhibitory efficacy on the growth of tumors* in vivo*, indicating that the anti-cancer effect of baicalin was not proportional to its dose in mice with colorectal cancer.

### Baicalin inhibited CRC cell metastasis *in vivo* and increased survival time of tumor-bearing mice

**Figure 9:**
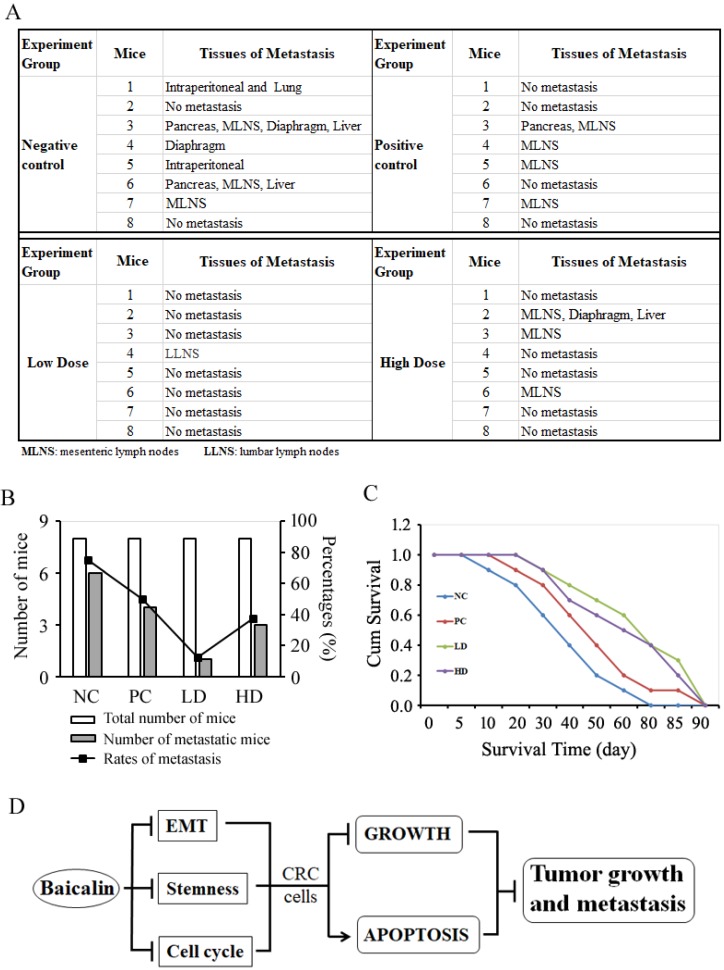
Baicalin inhibited colon tumor metastasis *in vivo* and increased survival time of tumor-bearing mice. **(A)** Thirty-two mice with orthotopic transplanted colon tumors were randomly divided into four groups and treated with negative/positive control reagents and low/high dose baicalin (as described in “Materials and Methods”) by intragastrical administration every day for four weeks, and then continued to be raised to two months. The metastasis of orthotopic tumor cells to other tissues and organs *in vivo* were checked after mice were sacrificed. Mice number and tissues of metastasis/no metastasis were recorded. **(B)** Data statistics of number of metastatic mice and percentages of metastasis in each group. **(C)** Twenty-one mice with orthotopic transplanted colon tumors were randomly divided into four groups and treated with negative/positive controls and low-/high-dose baicalin (as described in “Materials and Methods”) by intragastrical administration every day for four weeks, and then continued to be raised to death. The average survival days of mice in each group and the percentage of prolonged survival time of mice in baicalin and 5-FU groups (compared with negative control group) was calculated and presented. **(D)** Schematic diagram of integrated efficacy of baicalin in CRC cells *in vitro* and *in vivo*.

To study the effects of baicalin on the tumor metastasis *in vivo* and mice survival, the orthotopic transplanted colon tumor mice were treated with low-/high- doses of baicalin and negative/positive controls, which was the same as the tumor growth inhibition experiments; and then mice were raised for tumor metastasis assay and survival assay as described in materials and methods. From the metastasis assay data, we found that baicalin treatment significantly inhibited colorectal tumor metastasis *in vivo* by compared with negative control, and the metastasis-targeted tissues mainly included mesenteric lymph nodes (MLNS), liver, pancreas, diaphragm, intraperitoneal and lumber lymph nodes (LLNS) (Fig. 9A). The number of mice with tumor metastasis and the rates of mice with tumor metastasis in total mice were significantly less and lower in low-dose group (LD group) than those in both high-dose group (HD group) and positive control group (PC group); and the anti-tumor efficacy of low-dose baicalin was better than those of high-dose baicalin and positive control drug (Fig. 9B).

The mice survival assay (Kaplan-Meier analysis) showed that the average survival time of mice in baicalin treatment groups (including LD group and HD group) and PC group was longer than that of mice in NC group (Fig. 9C). The average survival time of mice in LD group were a little longer than that of mice in HD group (Fig. 9C). These data also indicated that the dose of baicalin *in vivo* was not the higher the better in anti-tumor metastasis and prolonging survival time of mice.

## Discussion

Although baicalin has been reported to possess the anti-cancer functions 19, its detailed molecular mechanism in CRC cells remains unclear and needs to be further investigated. Our data elucidated that baicalin treatment in CRC cells could down-regulate the levels of cell cycle-related proteins, EMT-related proteins and stemness-related proteins, and up-regulate the levels of cell epithelial markers and apoptosis-related proteins, and then resulted in cell cycle arrest, EMT inhibition, stemness weakness and apoptosis. Finally, the integrated effects of baicalin could result in the inhibition of growth and metastasis of CRC cells *in vitro* and *in vivo* (Fig. 9D).

TGF-β1, one of the members of the transforming growth factor family, promotes cell migration and invasion in many types of cancers by enhancing EMT 29, 30. In CRC cells, we found that baicalin treatment inhibited both endogenous and exogenous TGF-β1-induced EMT by suppressing TGF-β/Smad signaling pathway. It indicated that baicalin could inhibit CRC cell migration and invasion by inhibiting EMT *in vitro* and *in vivo*. The detailed molecular mechanisms for baicalin to inhibit TGF-β1/Smad signaling pathway still needed to be further studied.

Cancer cells with/without stem cell characteristics (stemness/non-stemness cancer cells) are important in the growth and development of tumors; stem cell characteristics (stemness) of cancer cells is particularly important and plays a key role in cancer metastasis, recurrence and drug resistance in many types of cancers in clinic 27, 28. A good medical compound (or drug) for cancer therapy should possess the efficacy which could inhibit both non-stemness and stemness cancer cells. Baicalin is a natural flavonoid compound isolated from *Scutellaria baicalensis Georgi*, a dicotyledon and Labiatae plant. Our *in vivo* and *in vitro* studies showed that baicalin could inhibit cell growth and induce apoptosis in both adherent-cultured CRC cells and suspensive-cultured CRC cell spheres, suggesting that baicalin was the compound which possessed the inhibitory efficacy in both non-stemness and stemness CRC cells. It implied that baicalin might be a good candidate for colorectal cancer treatment in clinic by its advantages in inhibiting both non-stemness and stemness CRC cells.

Apoptosis is an inevitable event in cancer radiotherapy and chemotherapy in clinic. In terms of the critical role of p53 protein in determining cell growth and apoptosis, there were two signal pathways which were responsible for inducing cell apoptosis, p53-dependent and -independent pathways 31, 32. Although wild type p53 was expressed in HCT116 and RKO cell lines 33, we still found that p53 protein levels had no significant changes with the baicalin treatment in both CRC cell lines. In addition, our data showed that the levels of c-Caspase 8, c-Caspase 9 c-Caspase 3 and c-Parp-1 were increased by treated baicalin. It indicated that the molecular mechanism of baicalin-induced CRC cell apoptosis was p53 independent pathway.

From our results, the *in vitro* inhibitory efficacy of baicalin on CRC cells was in a concentration dependent manner, while the in vivo inhibitory efficacy of low-dose baicalin was a little better than that of high-dose baicalin. This difference indicated that high concentration of baicalin *in vivo* might have some side effects or other unknown adverse effects and then result in its reduced inhibition on tumor growth and metastasis. The further studies to figure out the different effects of baicalin between *in vitro* and *in vivo* and its molecular mechanisms might be great favorable to the usage of baicalin in clinic in the future.

By summarizing our results, *in vitro* and *in vivo* experimental data obviously showed that baicalin-induced growth and metastasis inhibition in CRC cells (HCT116 and RKO cell lines) was a kind of multiple molecular suppression mode via inhibiting cell cycle/EMT/stemness, inducing cell apoptosis and regulating multiple signal pathways.

## Supplementary Material

Supplementary figure.

## Abbreviations

CRC: colorectal cancer
TGFβ: transforming growth factor-β
NF-κB: nuclear factor kappa-B
MAPK: mitogen-activated protein kinase
PBS: phosphate buffer saline
DMSO: dimethyl sulfoxide
PDGF: Platelet derived growth factor
CCK-8: cell counting kit-8
HRP: Horseradish peroxidase
MTT: methyl-tetrazolium
XIAP: X-linked inhibitor of apoptosis protein
FITC: fluorescein isothiocyanate
RIPA: radio immunoprecipitation Assay
