# Supplementary material for: Inhibiting EMT, stemness and cell cycle involved in baicalin-induced growth inhibition and apoptosis in colorectal cancer cells
Source: J Cancer. 2020 Feb 10;11(8):2303–17. doi: 10.7150/jca.37242 (PMC7052934; doi:10.7150/jca.37242)
Supplement: Supplementary file 1 — Supplementary figure. [file jcav11p2303s1.pdf]

Fig. S1

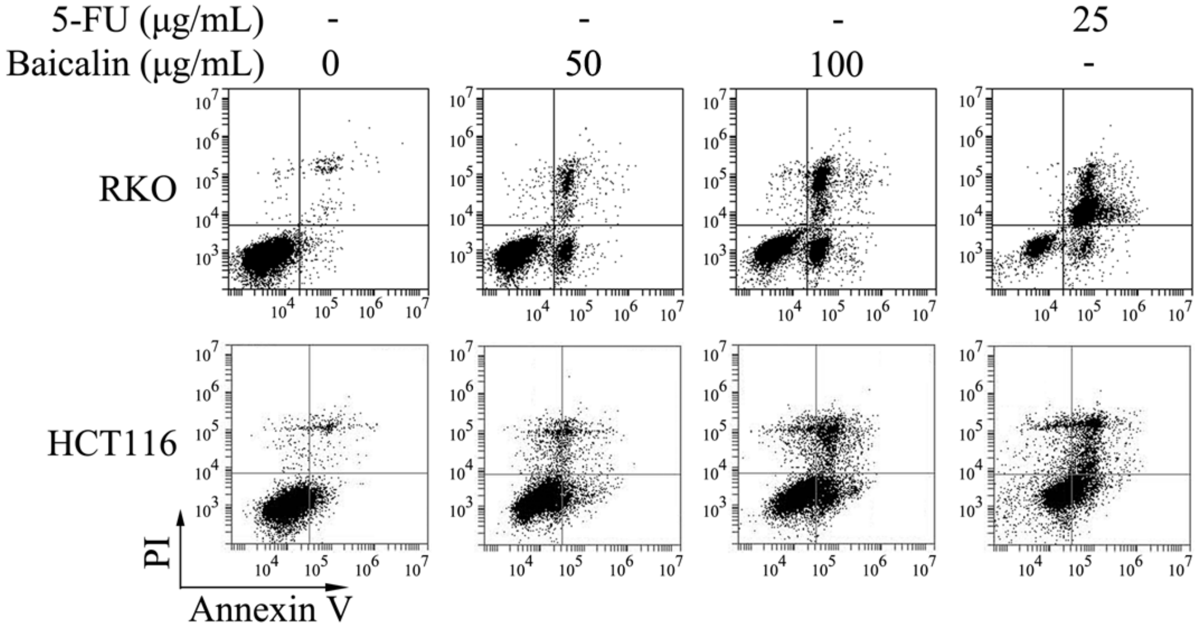

Figure S1. RKO and HCT116 cells which treated with 0, 50 and 100 μg/mL of baicalin and 25 μg/mL of 5-FU for 48 h were assayed for apoptosis by flow cytometry with Anexin V / propidium iodide double-staining. The apoptotic cells increased with increase of treated concentration of baicalin. The positive control was the cells treated with 25 μg/mL of 5-FU.
